# Microbial and Edaphic Responses to Invasion by *Ageratina adenophora* : Implications for Ecosystem Management

**DOI:** 10.1002/ece3.72983

**Published:** 2026-04-09

**Authors:** Yuqing Ma, Xiyu Zhang, Lei Huang, Qiangwei Wang

**Affiliations:** ^1^ Ministry of Agriculture Key Laboratory of Molecular Biology of Crop Pathogens and Insects, Zhejiang Key Laboratory of Biology and Ecological Regulation of Crop Pathogens and Insects, Institute of Pesticide and Environmental Toxicology Zhejiang University Hangzhou China

**Keywords:** bacterial community structure, co‐occurrence network analysis, plant–soil interactions, rhizosphere‐bulk soil differentiation, soil nitrogen dynamics, soil nutrient cycling

## Abstract

*Ageratina adenophora* is one of the most invasive and ecologically destructive plant species in southwestern China. Although its above‐ground competitive strategies have been widely studied, less is known about how it alters below‐ground soil processes and microbial communities that may in turn facilitate its spread. This study integrates both rhizosphere and non‐rhizosphere soils and applies co‐occurrence network analysis to characterize microbial responses to *A. adenophora* invasion. To assess the impact of *A. adenophora* on soil ecology, we compared soil physicochemical properties and bacterial communities between invaded and non‐invading sites. Using 16S rRNA high‐throughput sequencing and bioinformatic network analysis, we evaluated changes in microbial composition, abundance and interactions under invasion conditions. The soil moisture content was significantly reduced in areas populated with *A. adenophora,* especially in rC (58.84%) and nrC (65.55%), while the concentrations of NO_3_^−^‐N (especially in nrB, 221.52%) and NH_4_^+^‐N (especially in rD, 736.88%) were significantly elevated. The structure of the microbial community shifted markedly, with soil moisture and NH_4_^+^‐N identified as dominant factors shaping bacterial assemblages. Microorganisms that are significantly affected include *Proteobacteria*, *Actinobacterota*, and *Acidobacteriota*. These changes are likely to create favorable feedbacks that enhance *A. adenophora* 's invasive success. Our findings reveal that *A. adenophora* not only competes above ground, but also modifies soil conditions and microbial networks to reinforce its invasion. By integrating rhizosphere–non‐rhizosphere comparisons with microbial network analysis, this study provides a more detailed understanding of how *A. adenophora* alters soil microbial communities. These results emphasize the need to integrate soil microbial dynamics and nutrient feedbacks into the management of invasive species. Restoration strategies that regulate soil nitrogen levels and moisture may help suppress invader dominance and restore ecosystem function.

## Graphical abstract

This study investigates how the invasive plant *Ageratina adenophora* alters soil properties and microbial communities to facilitate its spread. The findings show that invasion is associated with reduced soil moisture, increased nitrogen levels, and significant shifts in microbial composition, suggesting feedback mechanisms that enhance its invasiveness.

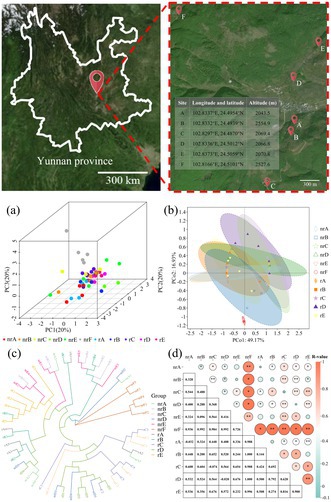

## 1 Introduction

Biological invasions are a leading threat to biodiversity and ecosystem stability worldwide. Invasion of alien species has garnered significant attention within the field of ecology. Through their distinctive biological traits and exceptional adaptability, nonnative species can rapidly colonize ecological niches in new environments, thereby exerting substantial and often profound effects on local ecosystems (Ji et al. 2014), including impacts on plant, insect, and microbial diversity (Gioria and Osborne 2014; Jiang et al. 2017; Kong et al. 2017). *Ageratina adenophora* , a weed native to Mexico, has become one of the most aggressive invaders in Asia and beyond. It is now widely distributed across multiple countries on several continents, including Asia, Africa, the Americas, and Oceania (Lu et al. 2005). Rapid spread of *A. adenophora* can be attributed to its allelopathic advantage and its significantly higher seed germination rate compared to many native plant species (Tripathi et al. 2018; Zhang et al. 2014).

In China, more than 30 million acres of arable land have been invaded by *A. adenophora* (Sun et al. 2018), primarily in the southern Yunnan, Guizhou and Sichuan provinces. The invasion is steadily advancing northward at a rate of approximately 20 km per year (Wang et al. 2017). *A. adenophora* reduces native plant diversity, weakens forest regeneration, and impacts crop yields. For example, studies have shown that *A. adenophora* can inhibit root growth in rice (Yang et al. 2008). Furthermore, Thapa et al. (2020) reported that *A. adenophora* suppresses the growth of native tree species such as *Schima wallichii* and *Alnus nepalensis* , and alters the structure of the vegetation in alder and oak forests in the central Himalayas (Rao et al. 2025). These hazards have also had a significant impact on the local economy.

Furthermore, compounds found in *A. adenophora* leaves have demonstrated hepatotoxicity in mice, with an LD_50_ of 926 mg/kg body weight (Ouyang et al. 2014). These toxins also affect animal organs and immune systems, posing a risk to livestock. It was reported to induce splenic and intestinal toxicity, along with a wide range of inflammatory responses (Ren et al. 2021; Fu et al. 2018; Cui et al. 2021).

The impact of *Ageratina adenophora* on soil properties is significant and ecologically concerning, as it directly reflects the plant's potential to degrade environmental quality. Invasion alters soil structure, leading to reduced fertility, acidification, and even salinization (Zhang and Wang 2010).

Previous research has demonstrated substantial alterations in soil nutrient profiles in areas invaded by *A. adenophora*. For example, Niu et al. (2007) reported higher levels of soil organic carbon, NO_3_^−^‐N, NH_4_^+^‐N, available phosphorus and potassium at heavily invaded sites, while the total potassium and pH values were lower than those of uninvading areas. Similarly, Kong et al. (2017) found that compared to soils under native vegetation, organic carbon and NO_3_^−^‐N concentrations were significantly elevated in soils affected by *A. adenophora* .

Soil microorganisms, as key drivers of nutrient cycling and soil structure formation, are particularly sensitive to environmental disturbances. Several studies have examined microbial responses to *A. adenophora* invasion. Liu et al. (2019), for example, observed significant shifts in the concentration and taxonomic composition of bacterial communities of the rhizosphere across different invasion intensities. They identified the potassium, organic matter content, invertase and urease activities available in the soil as the main factors shaping the structure of the microbial community.

The mechanisms underlying the interactions between plants and soil are complex and often subtle, making them difficult to fully capture with traditional methods. Recent advances in high‐throughput sequencing, particularly 16S rRNA gene sequencing combined with bioinformatics, offer powerful tools to unravel the dynamics, structure and links of microbial communities to environmental variables. This method allows detailed profiling of microbial shifts at the genetic level (Felske and Akkermans 1998; Chang et al. 2021; Wang et al. 2023).

In this study, we hypothesized that invasion by *A. adenophora* alters soil physicochemical properties and microbial community composition, with stronger effects in the rhizosphere than in non‐rhizosphere soils. To test this hypothesis, we used 16S rRNA sequencing and bioinformatic analysis to investigate differences in soil properties between areas invaded with *A. adenophora* and non‐invaded sites. We also compared rhizosphere and non‐rhizosphere soils within invaded sites. Our findings contribute to a more nuanced understanding of the ecological consequences of *A. adenophora* invasion and provide a scientific foundation for future research on invasive plant–soil–microbe interactions.

## 2 Materials and Methods

### 2.1 Experimental Design

**FIGURE 1:**
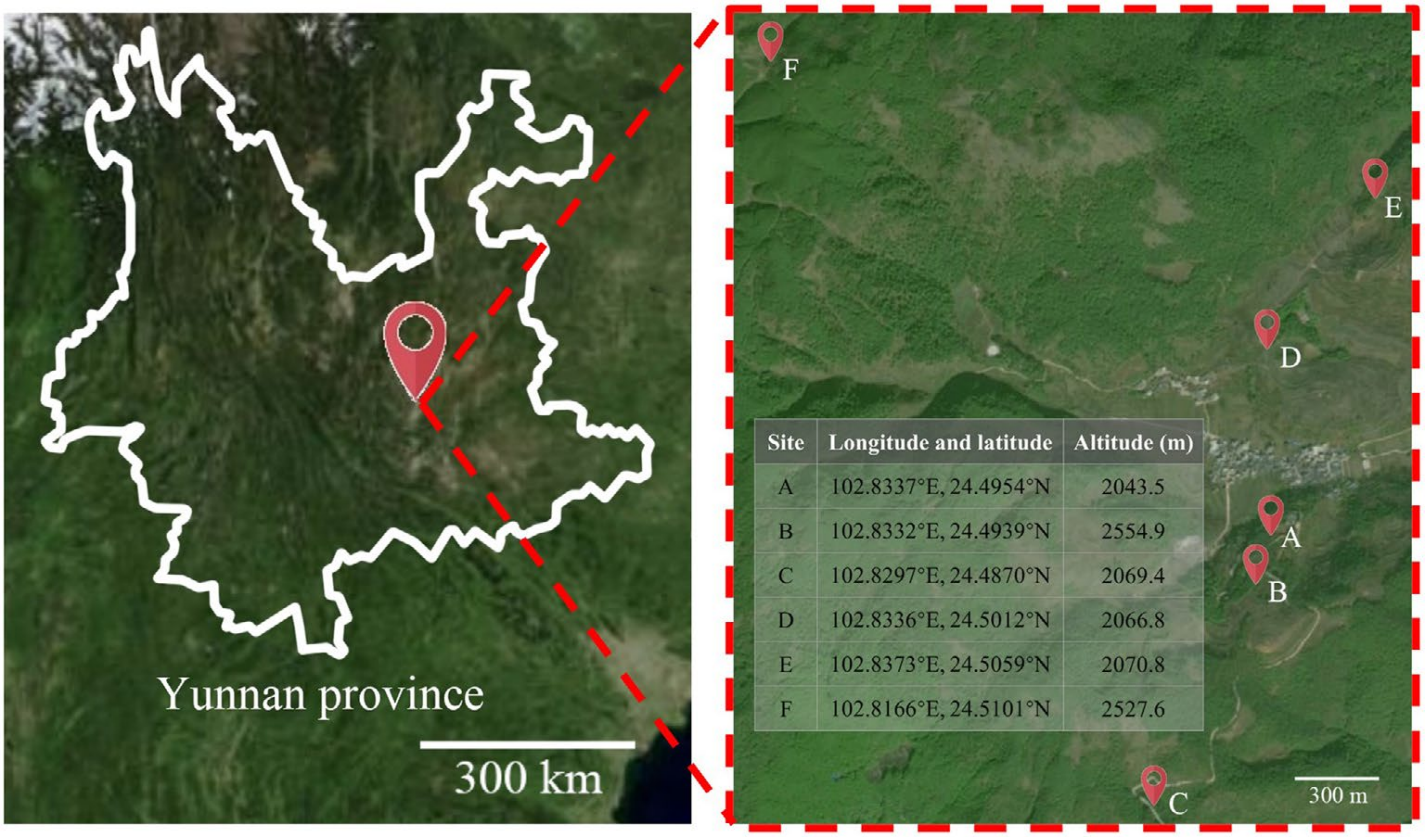
Sampling sites and field plot information in Yunnan Province, China. (a) Geographic location of the study area in Yunnan Province. (b) Satellite image of the sampling plots showing six sites (A–F). Sites A–E represent *Ageratina adenophora* –invaded plots, whereas site F represents the non‐invaded control. The table shows the longitude, latitude, and altitude of each site. Rhizosphere soil samples are labeled as rA–rE, and non‐rhizosphere soil samples are labeled as nrA–nrF.

*A. adenophora* and soil were collected on 17 November 2024, in Sanbaimu Village, Jiangchuan District, Yuxi City, Yunnan Province, China. The predominant type of vegetation in the area is shrubland. Five plots of *A. adenophora* with varying coverage rates and invasion durations exceeding 5 years were selected. Each plot covers an area of approximately 36 m^2^, and samples were collected using a five‐point sampling method within each plot, with each individual sampling area measuring approximately 1 m^2^. The intact Within each plot, three rhizosphere soil samples (attached to roots, r) and three non‐rhizosphere soil samples (surface soil not in direct contact with roots, nr) were collected using a five‐point sampling method. Non‐invaded soils from nearby areas were collected as controls. Brush was used to brush the soil adhered to the surface of the roots. Detailed information is provided in Figure 1. Soil samples were sieved in situ to remove nonsoil components, such as stones and insects. After processing, the samples were stored in sterilized bags and kept on ice for transportation to the laboratory. Once the soil samples were taken to the laboratory, it needed to be frozen with liquid nitrogen. Then store at −80°C.

### 2.2 Determination of the Soil Property

#### 2.2.1 Determination of the Moisture Content

Place 50 g of soil in a weighing box to calculate the total weight. Open the box cover and place it in an oven. Dry at 85°C for 72 h, then cool for 30 min and weigh. Repeat 3 times for each sample and calculate the moisture content.

#### 2.2.2 Determination of the pH Value

Contact 20 g of soil with 25 mL of ultrapure water and soak for 24 h, then use a pH meter (Mettler Toledo, Shanghai, China) to measure the pH value. Repeat 3 times for each sample.

#### 2.2.3 Determination of the NO_3_^−^ ‐N Content

Weigh 50 g of soil sample, add 0.5 g of CaSO_4_ · 2H_2_O (Sigma, St. Louis, USA) and 250 mL of ultrapure water, shake for 10 min, then take 25 mL of supernatant and add 0.05 g of CaCO_3_ (Sigma, St. Louis, USA). Dry with steam in a water bath and set aside. Take 10 μg/mL of the standard NO_3_^−^‐N solution (HACH, Loveland, USA), 0, 1, 2, 5, 10, 15, and 20 mL, respectively, and evaporate them in a water bath to dryness. Cool all evaporated samples and then add 2 mL of phenol disulfonic acid reagent. After 10 min, add 20 mL of ultrapure water. After mixing 1:1 NH_4_OH, add ultrapure water to make up to 100 mL. Measure the absorbance at 405 mm using a microplate reader (Thermo, Waltham, USA) and plot a standard curve to calculate the regression equation. For soil samples, after measuring absorbance, calculate the NO_3_^−^‐N content based on the standard curve. Repeat 3 times for each sample.

#### 2.2.4 Determination of the NH_4_ ^+^‐N Content

Take 0, 2, 4, 6, 8, and 10 mL of 2.5 μg/mL NH_4_^+^‐N standard solution (HACH, Loveland, USA), adding to 10 mL of KCl solution (1 mol/L, Sigma, St. Louis, USA), respectively. Measure the absorbance at 620 mm and plot the standard curve to calculate the regression equation. Take 20 g of soil, add 100 mL of KCl solution (1 mol/L) and shake for 1 h. After the system becomes clear, use the upper clear solution as soil leachate for analysis. Add 5 mL of phenol and 5 mL of NaClO_3_ (Sigma, St. Louis, USA) to 10 mL of soil leachate, shake and stand for 1 h; then dilute to 50 mL with ultrapure water. Measure the absorbance at 620 mm and calculate the NH_4_^+^‐N content based on the standard curve. Repeat 3 times for each sample.

#### 2.2.5 Determination of the Available Phosphorus Content

Take 0, 1, 2, 3, 4, and 5 mL of standard phosphorus solution (5 μg/mL, HACH, Loveland, USA) and add 10 mL of NaHCO_3_ (0.5 mol/L, Sigma, St. Louis, USA), respectively. Measure the absorbance at 710 mm and plot the standard curve to calculate the regression equation. Weigh 2.5 g of dry soil, add 50 mL of 0.5 mol/L NaHCO_3_ (Sigma, St. Louis, USA), shake for 30 min, filter with phosphate‐free filter paper, add 35 mL of ultrapure and 5 mL of Molybdate—anthnony‐scandium (Sigma, St. Louis, USA) to 10 mL of filtrate, let the mixture stand for 30 min to detect the absorbance and calculate the content. Repeat 3 times for each sample.

### 2.3 16S rRNA Sequencing

Based on primer 341F (5′‐CCTAYGGGRBGCASCAG‐3′) and 806R (5′‐GGACTACNNGGGTATCTAAT‐3′), the 16S V3–V4 regions of the 16S bacterial rRNA in soil samples were amplified. The specific operation is as follows: Add 15 μL PassionHigh‐Fidelity PCR Master Mix, 0.2 μM primers, and 10 ng genomic DNA template to all PCR mixtures, perform the first denaturation at 98°C for 1 min, then repeat 30 cycles at 98°C (10 s), 50°C (30 s), and 72°C (30 s), and finally maintain at 72°C for 5 min. Perform magnetic bead purification on the PCR product, mix equally according to the concentration of the PCR product, mix thoroughly, detect the PCR product, and recover the target band. Construct a library, perform Qubit and qPCR quantification on the constructed library, and perform sequencing after the library has been qualified.

### 2.4 Bioinformatics Analysis

#### 2.4.1 Quality Control, ASV Denoising and Species Annotation

FLASH (Version1.2.11, http://ccb.jhu.edu/software/FLASH/) Software such as Cutadapt and Fastp (Version 0.23.1) were used to split the sequencing results and obtain tag sequences, which were then compared with the species annotation database (Silvadatabase, https://www.arb‐silva.de/for 16S/18S, Unitedatabase, https://unite.ut.ee/). For ITS, compare and detect chimeric sequences, and ultimately remove the chimeric sequences to obtain the final valid data. Additionally, use QIIME2 software for ASV denoising, species annotation, and constructing phylo‐genetic trees. Finally, the obtained data are homogenized using the sample with the smallest amount of data as the standard.

#### 2.4.2 Vennand Flower Diagram

Venn and Flower diagrams visually display common and unique information between different groups. Venn and Flower diagrams were produced in R with VennDiagram() function and in Perl with SVG function, respectively.

#### 2.4.3 Relative Abundance

The top 10 taxa of each sample at each taxonomic rank (phylum, class, order, family, gene, species) were selected to plot the distribution histogram of relative abundance in Perl through the SVG function.

#### 2.4.4 Alpha Diversity

To analyze the diversity, richness, and uniformity of the communities in the sample, alpha diversity was calculated from 5 indices in QIIME2, including observed species, Chao1, Shannon, Simpson, Pielou's evenness.

#### 2.4.5 Beta Diversity

Beta diversity analysis was used to evaluate the differences in samples in species complexity. It was calculated on the basis of weighted and unweighted unifrac distances in QIIME2. Then a heat map was created to display the unifrac distance between samples, which was performed in Perl.

Cluster analysis was preceded by principal component analysis (PCA), which was applied to reduce the dimension of the original variables using the ade4 package and the ggplot2 package with R software (Version 4.0.3).

Principal Coordinate Analysis (PCoA) was performed to obtain the principal coordinates and visualize them from complex and multidimensional data. A distance matrix of weighted or unweighted unifrac among samples was obtained before transformation to a new set of orthogonal axes, by which the maximum variation factor is demonstrated by the first principal coordinate, the second maximum one by the second principal coordinate, and so on. The PCoA analysis was displayed using the ade4 package and the ggplot2 package in the R software (Version 4.0.3).

The unweighted pair group method with arithmetic means (UPGMA) is a method based on the similarity or distance matrix between samples, which gradually merges the samples into a cluster and calculates the average distance of the new cluster. It is used to construct a clustering tree. In this study, we used NovoMagic (https://magic. novogene.com/) conduct relevant analysis.

#### 2.4.6 Analysis of Similarities (ANOSIM)

ANOSIM was used to compare within‐group similarity through a distance measure to test the null hypothesis that the average rank similarity between samples within a group is the same as the average rank similarity between samples belonging to different groups. In this study, it was analyzed using NovoMagic (https://magic.novogene.com/).

#### 2.4.7 PICRUSt Test

PICRUSt uses a machine learning approach to predict the functions present in microbial samples by aligning 16S rRNA sequences with sequences of known functional genes. It was used to predict the functional composition of soil samples and to further analyze differences in functionality between groups. NovoMagic (https://magic.novogene.com/) was used to perform the relevant analysis.

#### 2.4.8 Research on the Interaction Between Microorganisms and Environment

The Spearman algorithm is used to analyze microbial interaction networks. The analysis method is to calculate the correlation index for all samples, obtain the species correlation coefficient matrix, and remove the connections with the correlation coefficient < 0.6 and the nodes and the abundance less than 0.005%.

The Mantel test is used to analyze the correlation between environmental factors and microbial community data, and it also utilizes RDA analysis to reflect the relationship between microbial communities and environmental factors, obtain the relationship between environmental factors, samples and microbial communities, and analyze important environmental driving factors that affect sample distribution. Furthermore, by using the Spearman algorithm to analyze the correlation and significant differences between microorganisms and environmental factors, the above analysis was performed using NovoMagic (https://magic.novogene.com/).

#### 2.4.9 Statistical Analysis

All soil physicochemical parameters, including moisture content, pH, NO_3_^−^‐N, NH_4_^+^‐N and available phosphorus, were measured in triplicate for each sample. The results were expressed as mean ± standard deviation. Group comparisons were conducted to assess differences between rhizosphere and non‐rhizosphere soils, as well as between invaded and non‐invading sites. The data was first tested for normality using the Shapiro–Wilk test. For normally distributed data, independent samples *t*‐tests or one‐way ANOVA were used; for nonnormally distributed data, the Mann–Whitney *U* test or the Kruskal‐Wallis test was applied. All statistical analyzes were performed with the R software (version 4.0.3) and a *p*‐value of less than 0.05 was considered statistically significant. Statistical analyzes of 16s rRNA were performed using R software (Version 4.0.3) and STAMP. Differences in soil properties, alpha diversity indices and functional pathways were evaluated using the Wilcoxon rank sum test or one‐way ANOVA, depending on the normality of the data distribution. For comparisons among multiple groups, the Kruskal–Wallis test followed by Dunn's post hoc test was used. The LEfSe analysis was performed via NovoMagic to identify differentially abundant taxa, with an LDA score threshold of 3.5 and *p* < 0.05. Where applicable, multiple comparisons were corrected using the Benjamini–Hochberg FDR method.

## 3 Results

### 3.1 *A. adenophora* Changed Soil Properties

**FIGURE 2:**
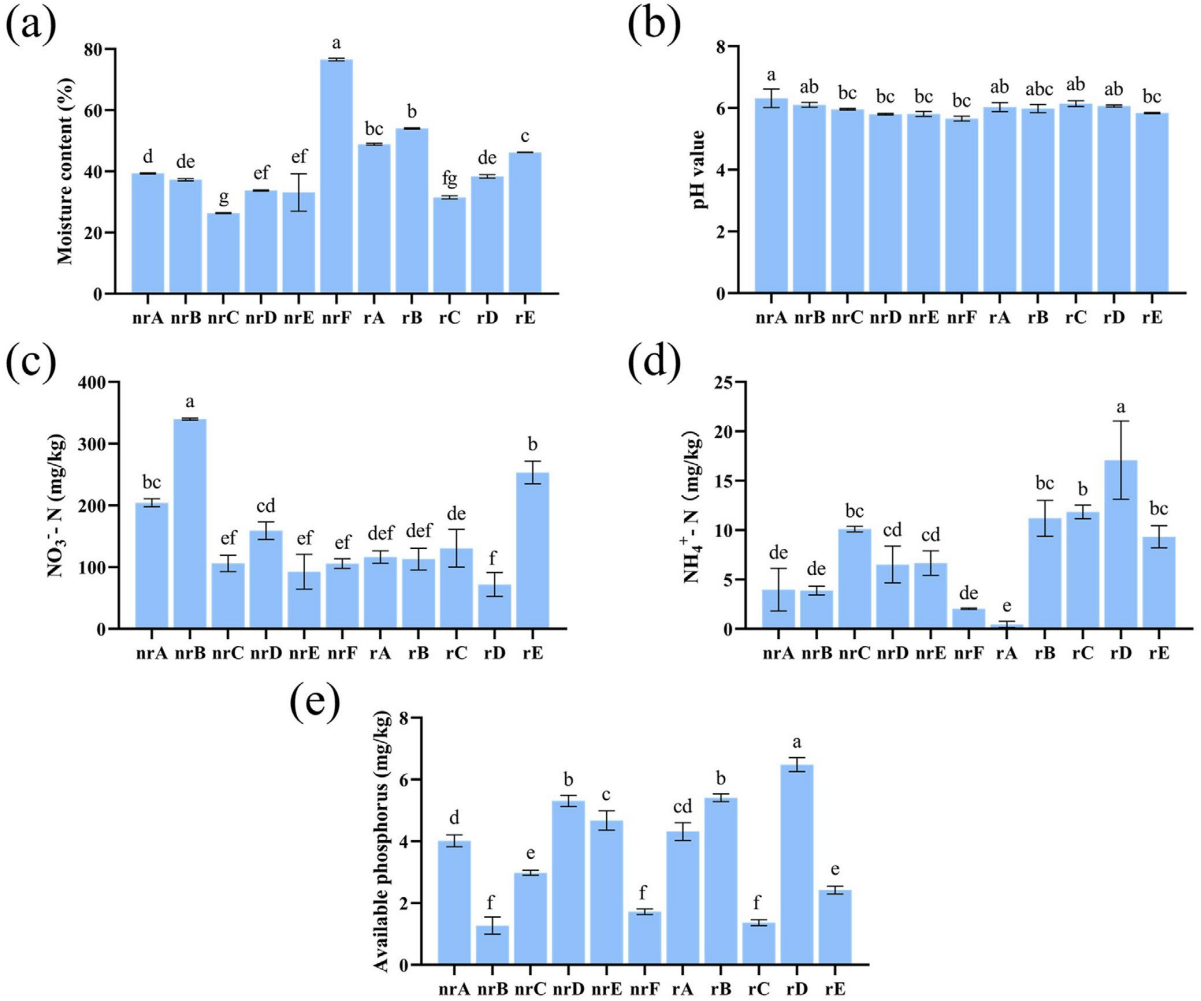
Effects of *Ageratina adenophora* invasion on soil physicochemical properties. (a) Moisture content, (b) pH, (c) NO_3_^−^‐N content, (d) NH_4_^+^‐N content, and (e) available phosphorus content in rhizosphere (r) and non‐rhizosphere (nr) soils from different sampling sites. Site F (nrF) represents the non‐invaded control. Data are presented as means ± SD Different lowercase letters above bars indicate significant differences among groups (*p* < 0.05).

The soil moisture content in both the invaded areas (rA, rB, rC, rD, rE, nrA, nrB, nrC, nrD and nrE) and non‐invaded areas (nrF) of *A. adenophora* is presented in Figure 2a. The results reveal that the soil moisture content in the invaded area is significantly lower than that in the non‐invaded area (*p* < 0.05). As shown in Figure 2b, *A. adenophora* also influences soil pH. Specifically, the pH of non‐rhizosphere soil in the invaded area (nrA) is significantly higher compared to non‐invaded area (*p* < 0.05), and other sites also exhibit an upward trend in pH. Figure 2c illustrates that the NO_3_^−^‐N content in the soils of the ABDE sites is higher compared to nrF (*p* < 0.05), indicating that *A. adenophora* contributes to an increase in the NO_3_^−^‐N content of the soil. While no significant differences were observed between region A and nrF, the NH_4_^+^‐N content in soils from BCDE regions was significantly greater than in areas without invasion (*p* < 0.05), suggesting that *A. adenophora* promotes an increase in the NH_4_^+^‐N content of the soil (Figure 2d). Finally, Figure 2e shows that the content of available phosphorus is significantly higher in soils from ABCDE sites compared to nrF (*p* < 0.05).

### 3.2 *A. adenophora* Disturbed the Community Structure of Soil Microorganisms

#### 3.2.1 *A. adenophora* Led to Changes in Soil Microbial OTUs

Through 16S rRNA sequencing of soil samples from various sites, a total of 55,278 OTUs were identified. The analysis of the rarefaction curve revealed that as the number of sequences increased, the curve approached a plateau, indicating a decrease in the rate of increase in newly identified OTUs and confirming that the depth of the sequencing was sufficient to capture most of the OTUs in the samples (Figure S1a). Additionally, the rarefaction curve shows that, compared to the other sites, the soil from the noninvasive area (nrF) has the lowest number of OTUs.

The OTUs of soil from the invasive and non‐invasive areas of *A. adenophora* were analyzed and the results are shown in Figure S1b, rA, rB, rC, rD, and rE have 8085, 8061, 7451, 7487, and 7790 OTUs, respectively, nrA, nrB, nrC, nrD, and nrE have 7261, 7405, 8358, 7708, and 8752 OTUs, respectively, nrF has 6264 OTUs. It can be easily observed that the number of OTUs in the invaded area of *A. adenophora* is higher than that in the noninvaded area. Further analysis of the differences in OTUs between rhizosphere and nonrhizosphere soils at the same site (Figure S1c) showed that there were 2471 identical OTUs in nrA and rA, with 4790 and 5614 unique OTUs, respectively; there were 2097 identical OTUs between nrB and rB, with 5308 and 5964 unique OTUs, respectively; there were 2600 identical OTUs between nrC and rC, with 5758 and 4851 unique OTUs, respectively; there were 2245 identical OTUs between nrD and rD, with 5463 and 5242 unique OTUs, respectively; nrE and rE have 1986 identical OTUs, with 6766 and 5804 unique OTUs, respectively. Our research also found that there are only 111 identical OTUs among nrA, nrB, nrC, nrD, nrE, and nrF, while other OTUs show differences. The specific number of differences is shown in Figure S1d. Additionally, a differential analysis of OTUs was performed on rA, rB, rC, rD, and rE, and the results are shown in Figure S1e. There are 438 OTUs that are common to the soils of the rhizosphere of each group.

#### 3.2.2 *A. adenophora* Affects the Richness of Soil Microorganisms

**FIGURE 3:**
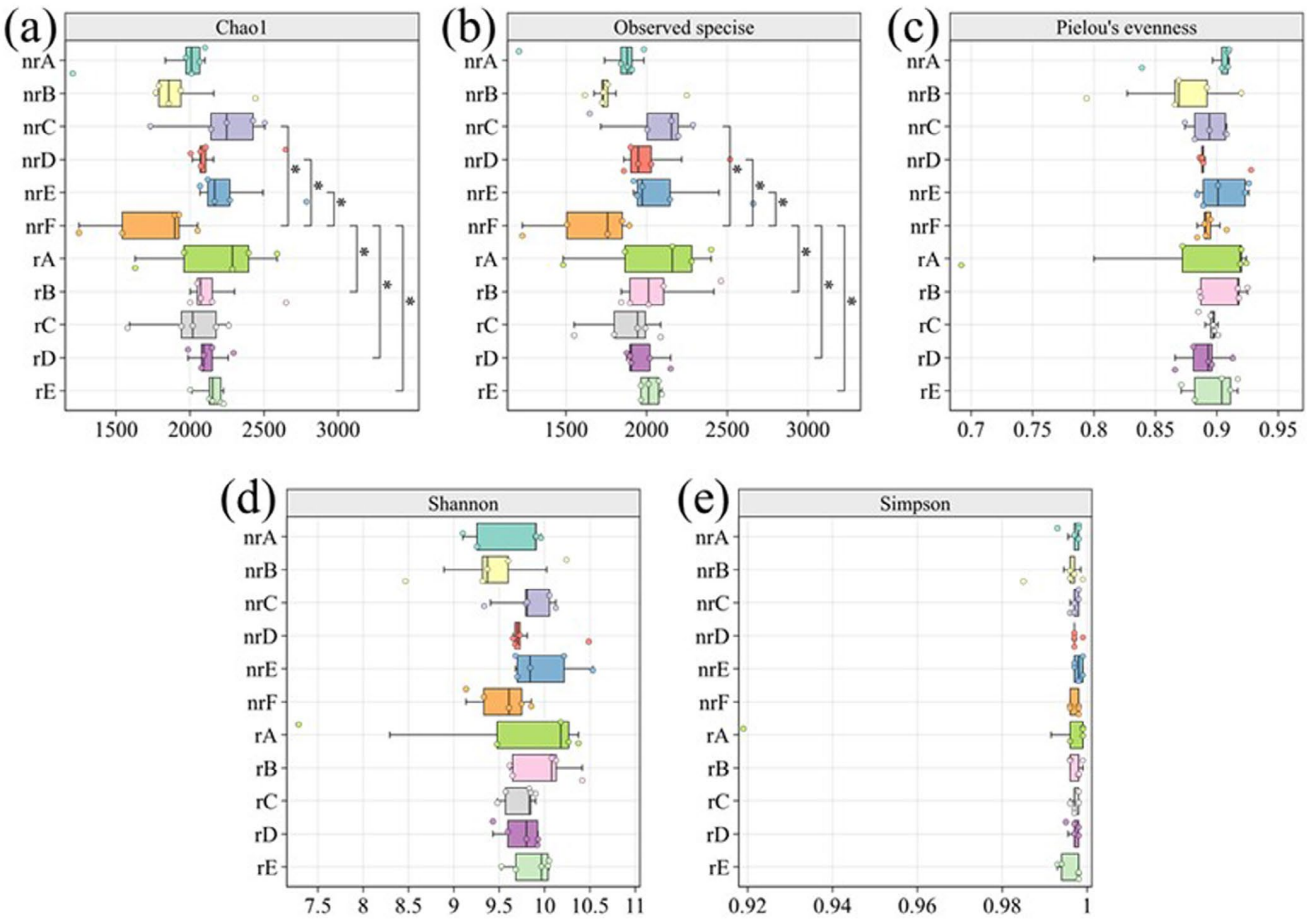
Alpha diversity of soil bacterial communities in invaded and non‐invaded soils. Boxplots showing (a) Chao1 richness, (b) observed species, (c) Pielou's evenness, (d) Shannon diversity, and (e) Simpson diversity for rhizosphere (r) and non‐rhizosphere (nr) soils across sampling sites. Site F (nrF) represents the non‐invaded control. Asterisks indicate significant differences between groups (*p < 0.05).

The results of the alpha diversity analysis are presented in Figure 3. Chao1, which estimates the total number of species in the sample, revealed that, compared to nrF, the Chao1 values were significantly higher at the sites of rB, rD, rE, nrC, nrD, and nrE (Figure 3a). Similarly, as shown in Figure 3b, the species richness observed in the soil of these sites was significantly greater than in nrF, indicating a difference in microbial richness between the invasive and non‐invasive areas of *A. adenophora* . However, Pielou's evenness, as well as Shannon and Simpson diversity indices (Figure 3c–e), suggest that there were no significant changes in microbial diversity and uniformity between soil samples from the various sites. Notable differences were also found when site nrF was compared to sites B, C, D, and E.

#### 3.2.3 *A. adenophora* Affects the Community Composition of Soil Microorganisms

**FIGURE 4:**
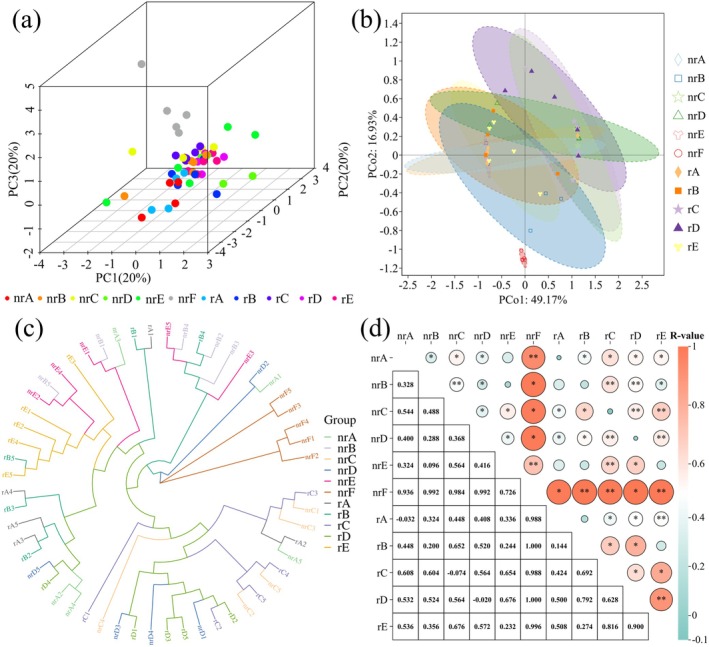
Beta diversity and clustering analyses of soil bacterial communities. (a) Principal component analysis (PCA) of bacterial community composition. (b) Principal coordinates analysis (PCoA) based on community dissimilarity. (c) UPGMA clustering tree showing relationships among samples. (d) ANOSIM results showing differences in bacterial community composition among groups. Rhizosphere soils are labeled rA–rE, non‐rhizosphere soils are labeled nrA–nrF, and nrF represents the non‐invaded control.

The results of the principal component analysis (PCA) are presented in Figure 4a. Spatial differences were observed between the nrF and the other sites, suggesting different variations in the composition of the soil microbial community between the invasive and noninvasive areas of *A. adenophora* . By using these two principal axes, approximately 60% of the variance in the data can be explained.

The results of the PCoA analysis are shown in Figure 4b. Compared to clustering within invaded areas of *A. adenophora* , the distance between nrF and the other sites was greater, indicating a distinct difference in soil microbial composition between invaded and non‐invading areas of *A. adenophora* . By using these two principal axes, 66.1% of the variance in the data can be explained.

The results of the UPGMA analysis are presented in Figure 4c. Compared to the branch distances between sites A, B, C, D, and E, the branch distance of nrF was significantly greater, with the nrF samples clustering within the same node. On the contrary, the clustering pattern of the other sites was more complex. These findings indicate that nrF exhibits significant differences from the other samples.

ANOSIM was also carried out to assess differences in microbial communities between soil samples (Figure 4d). The results revealed significant differences in the microbial composition between nrF and the other samples. However, no significant differences were observed in the microbial composition between rhizosphere and non‐rhizosphere soils within the invaded areas of *A. adenophora* .

### 3.3 Impact of *A. adenophora* on Soil Microorganisms

#### 3.3.1 *A. adenophora* Affects the Abundance of Soil Microorganism Species

Based on the results of the OTU annotation, microorganisms in the soil were classified into 42 phyla. As shown in Figure S2a, the top 10 most abundant phyla include *Proteobacteria*, *Actinobacteriota*, *Acidobacteriota*, *Gemmatimonadota*, *Chloroflex*i, *Myxococcota*, *Bacteroidota*, *Verrucomicrobiota*, *Crenarchaeota* and *Latescibacterota*. The relative abundance of these phyla in different soil sites is presented in Figure S2b. The *Proteobacteria* exhibited the highest relative abundance in the rhizosphere soil of rA, rB and rC, at 46.92% ± 12.84%, 45.37% ± 4.16%, and 46.61% ± 6.14%, respectively. The relative abundance of *Actinobacteriota* was significantly higher in rC and nrC, accounting for 25.25% ± 8.11% and 29.46% ± 9.10%, respectively. *Acidobacteriota* was the third most dominant group, with the highest relative abundance observed in nrF, at 29.43% ± 7.82%. The relative abundance of *Gemmatimonadota* was also relatively high in rDNA and NRD, at 21.34% ± 8.95% and 22.40% ± 7.31%, respectively. Furthermore, *Chloroflexi* showed a relatively high abundance in nrF and nrB, at 14.90% ± 5.95% and 17.00% ± 13.57%, respectively. As illustrated in Figure S2c, the invasion of *A. adenophora* significantly increased the abundance of *Gemmatimonadota* and *Bacteroidota*.

Among the 693 detected genera, *Proteobacteria* contains 267 genera, *Actinobacteria* includes 126 genera, *Gemmatimonadota* comprises 6 genera, *Chloroflexi* consists of 29 genera, *Myxococcota* has 22 genera, *Bacteroidota* includes 67 genera, *Verrucomicrobiota* contains 29 genera, and *Crenarchaeota* has 4 genera. The top 10 genera in terms of abundance include *Acinetobacter*, *Sphingomonas*, *Gemmatimonas*, *RB41*, *Flavobacterium*, *unidentified_TK10*, *Comamonas*, *MND1*, *Pseudonocardia*, and *Anaeromyxobacter*. The relative abundance of these genera at different sites is shown in Figure S2d. The results of the analysis of significant differences at the genus level revealed that the invasion of *A. adenophora* led to a significant increase in the abundance of *Sphingomonas*, while the abundance of *unidentified_TK10* and *Comamonas* was significantly reduced (Figure S2e).

#### 3.3.2 Screening Results of Differential Microorganisms

**FIGURE 5:**
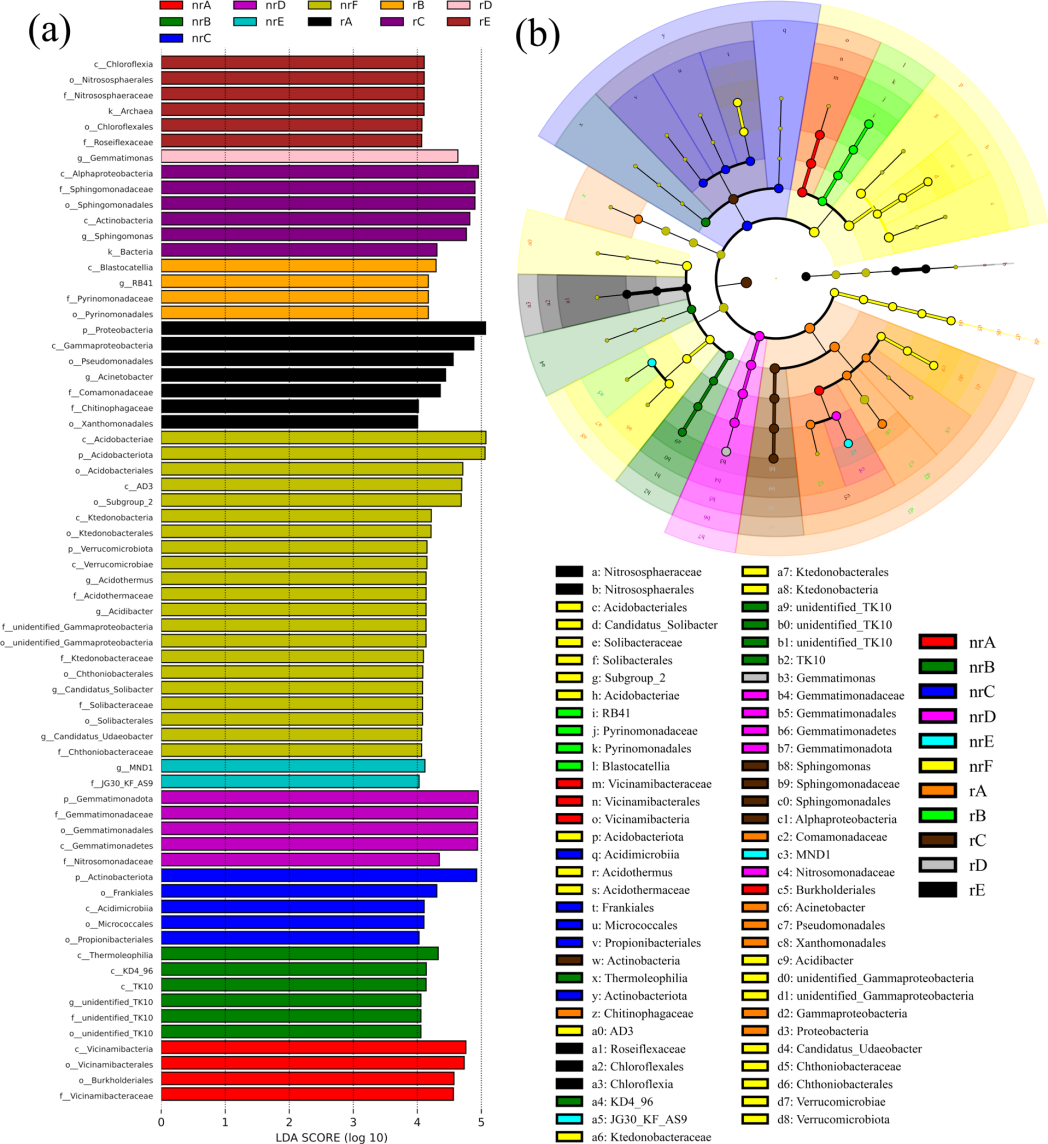
Differential bacterial taxa associated with *Ageratina adenophora* invasion. (a) LEfSe analysis showing bacterial biomarkers with LDA scores > 4 among different soil groups. Colored bars indicate taxa significantly enriched in the corresponding group. (b) Cladogram showing the phylogenetic distribution of differential taxa from phylum to genus level. Colored nodes indicate taxa with significantly higher relative abundance in the corresponding group, whereas yellow nodes indicate taxa with no significant difference.

Figure 5a presents microorganisms with LDA scores greater than 4, which are considered statistically significant biomarkers. The colored columns represent species with significant differences in abundance across different groups, while the length of each bar indicates the magnitude of the impact of these significantly different species. In the evolutionary branching diagram (Figure 5b), the circles, radiating from the center outward, represent taxonomic levels from phylum to genus. Each small circle at a given classification level corresponds to a specific taxon at that level, and the diameter of the circle is proportional to the relative abundance. We observed that, compared to other sites, nrF contains 21 biomarkers, significantly more than those found in other sites, suggesting substantial species differences between noninvasive and invasive areas of *A. adenophora* .

#### 3.3.3 *A. adenophora* Affects the Interaction Between Soil Microorganisms

Co‐occurrence networks were constructed at the genus level, and the results are presented in Figure S3. Significant differences in microbial adaptability were observed between the invasive area (Figure S3a) and the noninvasive area (Figure S3b). The dominant species in the invasive area include *Steroidobacterium*, *Pedomicrobium*, *Noviherbaspirillum*, *Jatrophihabitans*, *Pedocococcus*, and *Phytococcus*, which exhibit the closest associations with other species. In contrast, the dominant species in the noninvasive area include *Flavitalea*, *Altererythrobacter*, *Virgisporangium*, *Frankia*, and *Amnibacterium*. In addition to shifts in the dominant populations, we also found that, compared to the invasive area, the correlations between microorganisms in the noninvasive area are more tightly clustered. This suggests that the invasion of *A. adenophora* has disrupted the interrelationships among microbial communities.

### 3.4 *A. adenophora* Alters the Interaction Between Soil Microorganisms and Environmental Factors

**FIGURE 6:**
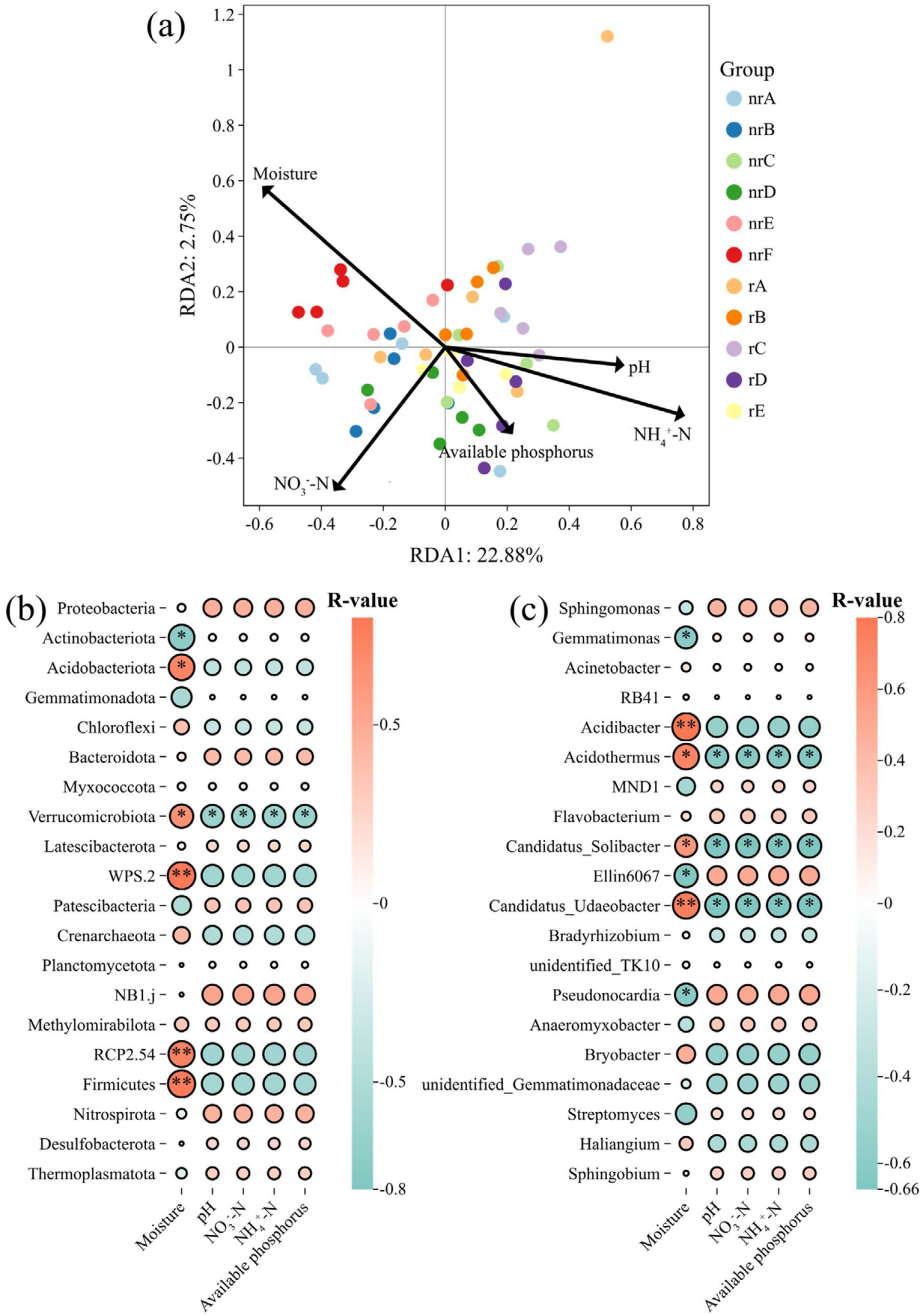
Relationships between soil bacterial communities and environmental factors. (a) Redundancy analysis (RDA) showing the effects of soil moisture, pH, NO_3_^−^‐N, NH_4_^+^‐N, and available phosphorus on bacterial community composition. (b) Spearman correlation heatmap between environmental factors and dominant bacterial phyla. (c) Spearman correlation heatmap between environmental factors and dominant bacterial genera. Circle color indicates the direction of the correlation, and circle size indicates the strength of the correlation. Asterisks indicate significant correlations (*p < 0.05; **p < 0.01).

The RDA analysis and the Mantel test were used to analyze the relationship between soil microorganisms and environmental factors. In Figure 6a, the arrows represent different environmental factors, while the colored dots correspond to soil samples from various sites. Among environmental factors, moisture (*p* = 0.010) and NH_4_^+^‐N (*p* = 0.010) were identified as the primary drivers of changes in soil microorganisms (*r* ^2^ = 0.4919). The Mantel analysis further confirmed that moisture (*r* = 0.5178, *p* = 0.013) and NH_4_^+^‐N (*r* = 0.1212, *p* = 0.018) are the main factors that influence soil microorganisms. These results suggest that *A. adenophora invasion* directly alters soil moisture and NH_4_^+^‐N content, driving changes in soil microorganisms.

The relationship between environmental factors and soil microorganisms was analyzed at both the levels of phylum (Figure 6b) and genus (Figure 6c). Spearman correlation analysis revealed that moisture, pH, NO_3_^−^‐N, NH_4_^+^‐N, and available phosphorus exert significant effects on soil microorganisms. At both the phylum and genus levels, moisture was found to have the most profound impact on microbial communities, significantly influencing the abundance of several species, including *Actinobacteriota*, *Acidobacteriota*, *Verrucomicrobiot*a, *Gemmatimonas*, *Acidobacter* and *Candidatus_Solibacter*. Furthermore, we identified *Verrucomicrobiota*, *Acidothermus*, *Candidatus_Solibacter*, and *Candidatus_Udaeobacter* as the most sensitive taxa in response to changes in environmental factors.

### 3.5 The Impact of *A. adenophora* on the Function of Soil Microorganisms

The top 35 functions in terms of relative abundance for the prediction functions are shown in Figure S4. Compared to other sites, the abundance of porphyrin and chlorophyll metabolism, carbon fixation pathways in prokaryotes, Fatty acid metabolism, Butanoate metabolism, Propanoate metabolism, Valine, leucine and isoleucine degradation, Pyruvate metabolism, Arginine and proline metabolism, lipid biosynthesis proteins, Methane metabolism, ABC transporters and transporters in nrF were significantly reduced, while the abundance of DNA repair and recombination proteins, Oxidative phosphorylation, Amino sugar and nucleotide sugar metabolism, Peptidases, Alanine, aspartate and glutamate metabolism, Transcription factors, Glycine, serine and threonine metabolism, Transcription machinery, General function prediction only significantly increased. This suggests that *A. adenophora* has modified the functional profiles of microorganisms in the soil. Additionally, we observed distinct functional differences between rhizosphere and non‐rhizosphere soils at the same site, highlighting that *A. adenophora* exerts differential effects on these two soil compartments.

## 4 Discussion

As an invasive species, *A. adenophora* poses a significant threat to the ecosystems of invaded areas due to its widespread distribution and rapid reproductive capacity. In this study, we observed that *A. adenophora* substantially alters soil properties, providing valuable information on its ecological adaptability, while also highlighting its potential detrimental effects on soil health and ecosystem functioning. Specifically, *A. adenophora* enhances the levels of NO_3_^−^‐N and NH_4_^+^‐N in the soil through mechanisms such as root exudates and residue decomposition (Niu et al. 2007). The increased organic matter content, including NH_4_^+^‐N, contributes to an imbalance in soil nutrient structure and leads to alterations in soil composition. These alterations in soil properties directly influence soil fertility and the diversity of plant communities, thus facilitating the further spread of *A. adenophora* (Jiang et al. 2022). Additionally, changes in soil structure can affect water infiltration and retention capacities, which can exacerbate soil erosion, a finding consistent with our results. This, in turn, poses significant challenges to agricultural productivity and the long‐term sustainability of natural landscapes. However, there is ongoing debate regarding the effects of *A. adenophora* on soil moisture content, and some studies suggest that its invasion may increase soil moisture levels (Kumar et al. 2021). This discrepancy indicates that the environmental impact of *A. adenophora* may vary between different habitats, soil type, season and elevation. Although this study did not find that *A. adenophora* invasion significantly alters soil pH, it contradicts the findings of Xiao et al. (2017), who reported a marked increase in soil pH after invasion. However, our study showed a similar upward trend, which may be attributed to the fact that the soil pH in the sampling area was already suitable for the growth of *A. adenophora*, thus preventing the secretion of more substances that could further alter the pH. In conclusion, changes in soil properties induced by *A. adenophora* not only pose a direct threat to soil health, but can also disrupt the stability and productivity of the entire ecosystem through a complex cascade of effects.

Microbial communities are integral components of soil ecosystems, playing essential roles in nutrient cycling, disease suppression, and maintenance of ecological functions (Zhu et al. 2021). This study demonstrates that the invasion of *A. adenophora* significantly alters the composition, structure, and interactions of soil microbial communities. These changes can result in the decline or loss of beneficial microorganisms, while promoting the proliferation of potentially harmful ones, thereby exerting profound effects on the health and functioning of soil ecosystems. For example, the function of *Acidobacteriota* involves nitrogen fixation, phosphorus solubilization, exopolysaccharide production, siderophore synthesis, and the production of plant growth hormones (Gonçalves et al. 2024, Flieder et al. 2021). A reduction in its abundance could alter the efficiency of nutrient conversion in the soil, potentially limiting plant growth and crop yields. Similarly, *Sphingomonas* has been reported to degrade organic pollutants and promote plant growth (Pan et al. 2024; Sultana et al. 2024). It can produce highly beneficial phytohormones, such as sphingan and gellan gum (Asaf et al. 2020). Our findings indicate that the invasion of *A. adenophora* significantly increases the abundance of *Sphingomonas*, suggesting that changes in the composition of the microbial community after its invasion could further support plant growth and spread. Furthermore, changes in the structure of the microbial community can impact soil carbon cycling and greenhouse gas emissions, indirectly influencing global climate change (Naylor et al. 2020). Results showed that, there is on significant differences in alpha diversity and community composition between rhizosphere and non‐rhizosphere soils. Potential reasons may include that *A. adenophora* , as an invasive alien species, conducts an “ecological cleansing” and “homogenization transformation” of the entire root zone soil through potent allelopathic effects and resource competition. This process selects for and shapes a relatively uniform microbial environment that is advantageous to the plant itself. Such a strategy reduces its dependence on the complex and variable native microbial communities in the new environment, lowers the difficulty of population establishment, and thereby facilitates its rapid expansion and dominance. Therefore, the impact of *A. adenophora* on soil microbial communities is not only crucial for maintaining soil ecological balance but is also intricately linked to broader ecological and environmental challenges, warranting further investigation and the implementation of effective management strategies.

This study not only confirms the significant impact of *A. adenophora* on soil properties and microbial communities, but also offers a novel perspective on the ecological mechanisms of invasive alien species and their potential threats to ecosystem services. The findings provide a scientific foundation for the development of effective strategies for managing invasive species. From a practical point of view, understanding how *A. adenophora* alters the soil environment can inform the design of targeted prevention and control measures, such as biological substitution, chemical control, or physical isolation, to mitigate its negative impact on ecosystems. For example, *Lolium perenne* (Poaceae), a perennial forage, has high economic and nutritional value. It has been used as a replacement control for *A. adenophora* (Shi et al. 2025). Further studies showed that, seven norsesquiterpenes isolated from the whole plant of *L. perenne* exhibited potent allelopathic effects on the growth of *A. adenophora* . Herbicides mainly composed of these compounds are being developed. Perhaps we can achieve the effect of inhibiting the growth of *A. adenophora* by targeted killing of bacteria that significantly inhibit its invaded. Furthermore, this study underscores the importance of soil health and microbial diversity, highlighting the need to strengthen the protection and management of soil ecosystems in the context of ecological restoration and sustainable agricultural practices. With the future in mind, modern technologies such as high‐throughput sequencing and isotope labeling will enable a deeper exploration of the interactions between *A. adenophora* and soil microorganisms, providing valuable insights that can support the development of more precise and effective management strategies.

## 5 Conclusion

Invasion by *A. adenophora* significantly alters soil physicochemical properties and microbial communities. Soil moisture decreases while NH_4_^+^‐N and NO_3_^−^‐N concentrations increase, creating conditions that may facilitate further invasion. Microbial community composition and interactions, particularly among *Proteobacteria*, *Actinobacterota*, and *Acidobacteriota*, are strongly affected, indicating feedbacks that reinforce the plant's ecological dominance. These findings highlight the importance of considering both soil nutrient dynamics and microbial interactions in invasive species management. Restoration strategies that regulate soil nitrogen levels, moisture, and microbial networks can help suppress *A. adenophora* and promote ecosystem recovery.

## Author Contributions

**Yuqing Ma:** conceptualization (equal), formal analysis (equal), investigation (equal), methodology (equal), writing – original draft (equal), writing – review and editing (equal). **Xiyu Zhang:** data curation (equal), formal analysis (equal), investigation (equal). **Lei Huang:** data curation (equal), formal analysis (equal), investigation (equal). **Qiangwei Wang:** conceptualization (equal), funding acquisition (equal), methodology (equal), methodology (equal), writing – review and editing (equal).

## Funding

This work was supported by the National Key Research and Development Program of China (2023YFC2604500) and the National Natural Science Foundation of China (Grant 22176173).

## Conflicts of Interest

The authors declare no conflicts of interest.

## Supporting information


**Figure S1:** ece372983‐sup‐0001‐FigureS1‐S4.docx.

## Data Availability

The raw sequencing data supporting the conclusions of this study have been deposited in the National Center for Biotechnology Information (NCBI) Sequence Read Archive (SRA) under BioProject accession number PRJNA1264738.
