# Supplementary material for: Microbial and Edaphic Responses to Invasion by Ageratina adenophora : Implications for Ecosystem Management
Source: Ecol Evol. 2026 Apr 9;16(4):e72983. doi: 10.1002/ece3.72983 (PMC13063388; doi:10.1002/ece3.72983)
Supplement: Supplementary file 1 — Figure S1: ece372983‐sup‐0001‐FigureS1‐S4.docx. [file ECE3-16-e72983-s001.docx]

**Supporting information**

**Microbial and Edaphic Responses to Invasion by *Ageratina adenophora*: Implications for Ecosystem Management**

| 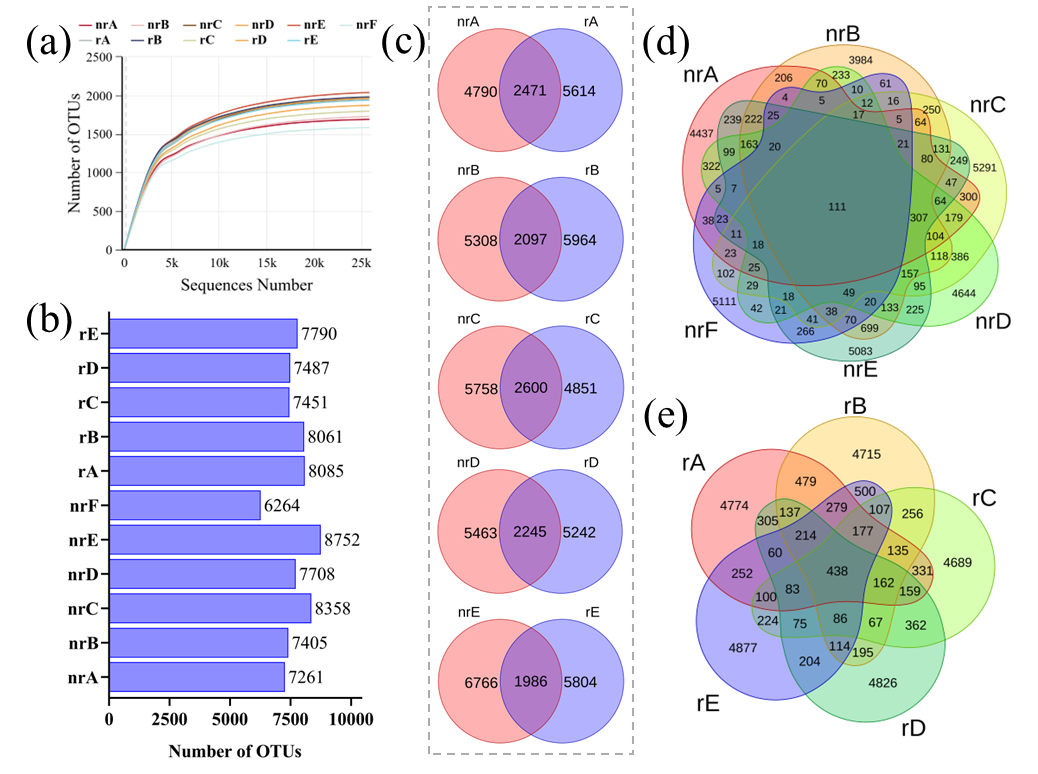 |
| --- |
| **Figure S1.** Number of observed OTUs in rhizosphere and non-rhizosphere soils at different sites. OTUs were clustered at 97% sequence identity based on ASVs generated by QIIME2. |

| 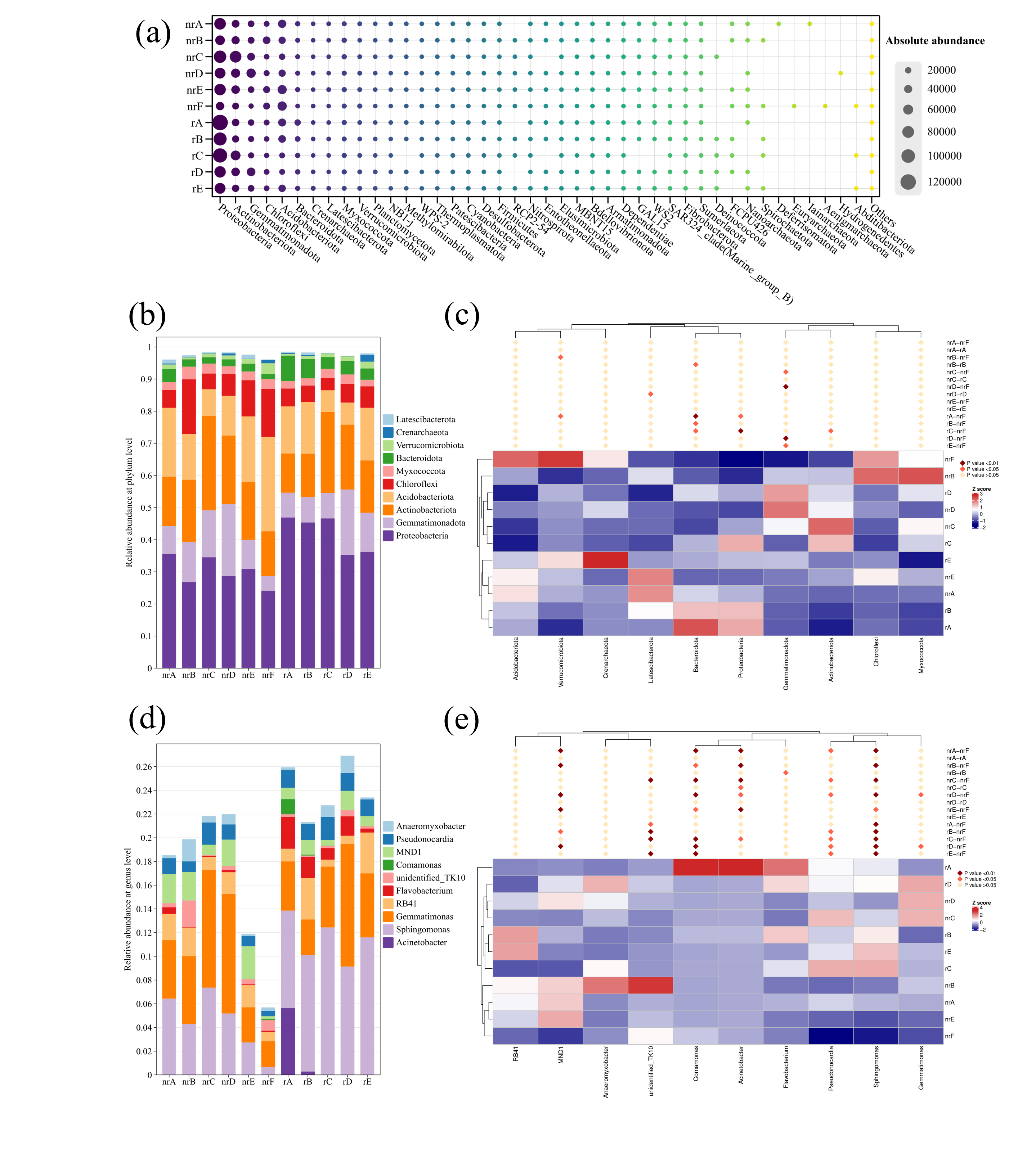 |
| --- |
| Figure S2. Relative abundance of dominant bacterial taxa at phylum and genus levels in rhizosphere and non-rhizosphere soils. Top 10 most abundant taxa are shown. Colors represent different taxonomic groups. |

| 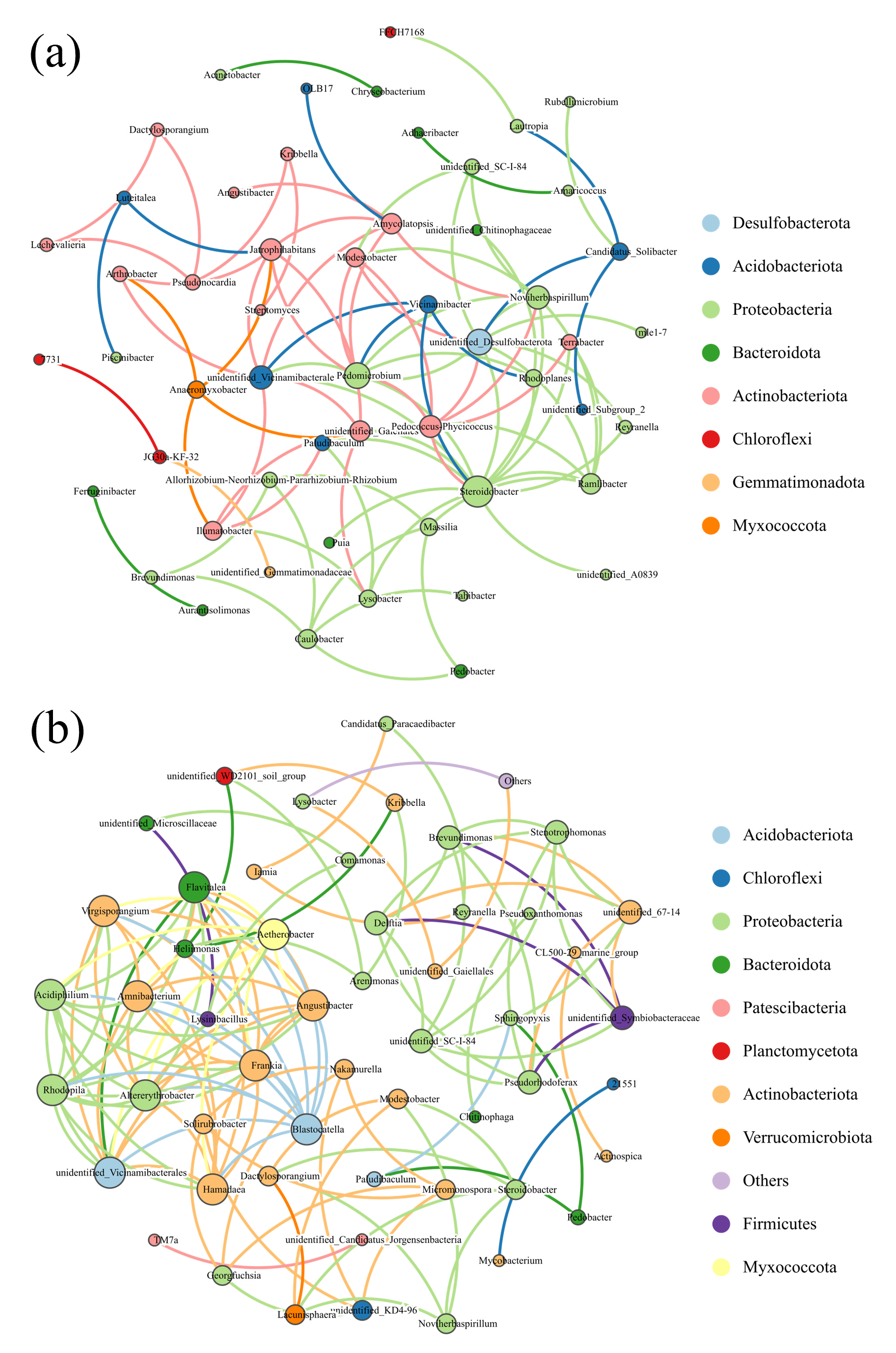 |
| --- |
| Figure S3. Co-occurrence network of soil bacterial taxa before (b) and after (a) A. adenophora invasion. Networks constructed using Spearman correlation coefficients > 0.6 and abundance threshold of 0.005%. Node size reflects taxon abundance; edge thickness indicates correlation strength. |

| 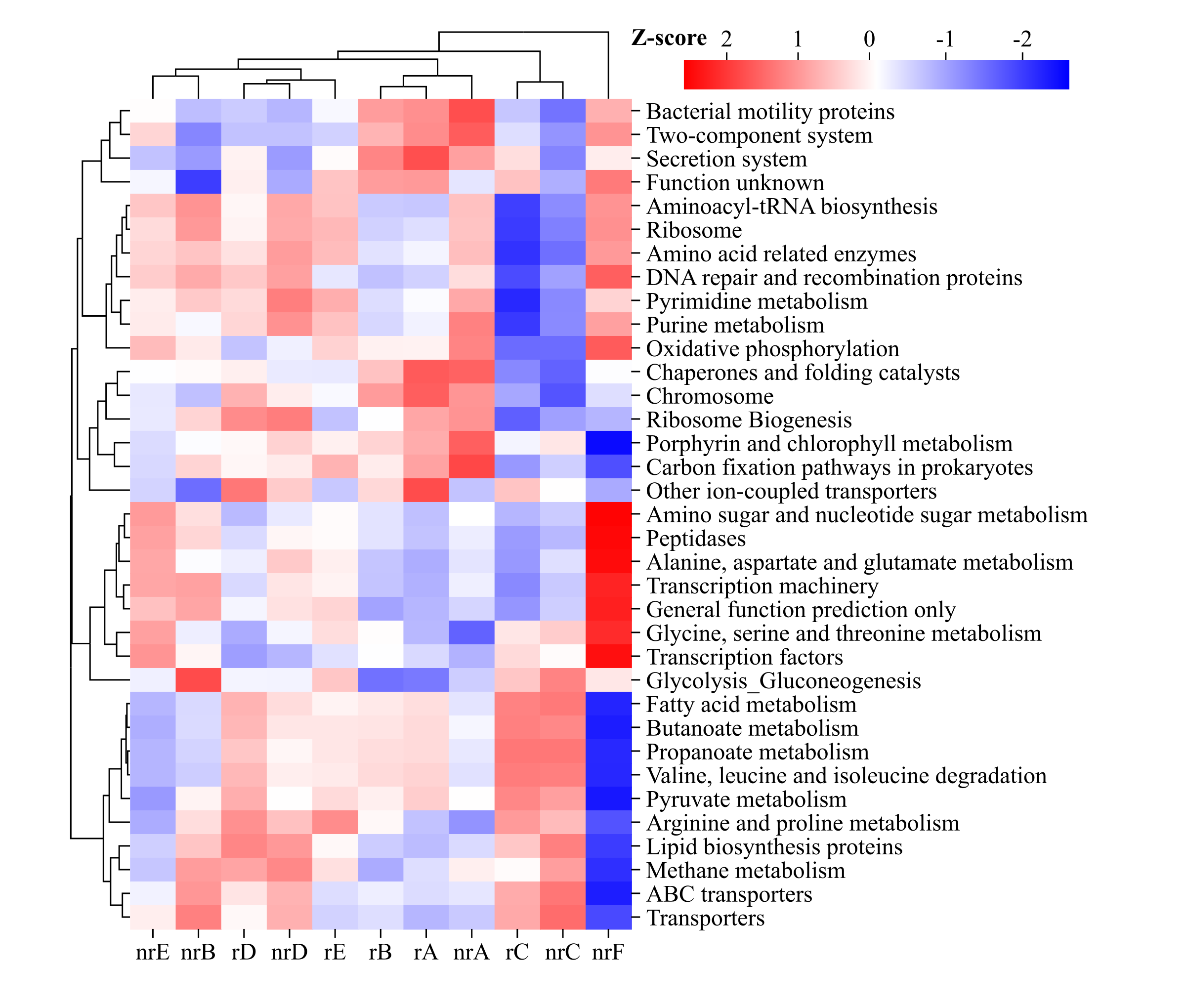 |
| --- |
| Figure S4. Functional prediction of bacterial communities using PICRUSt based on 16S rRNA gene sequencing. Relative abundance of top 20 predicted KEGG pathways is shown across sample types. Error bars represent standard deviation across replicates. |
